# Development, validation and greenness assessment of a new electro-driven separation method for simultaneous analysis of cefixime trihydrate and linezolid in their fixed dose combination

**DOI:** 10.1186/s13065-023-01049-3

**Published:** 2023-10-04

**Authors:** Maha R. Habeeb, Samir M. Morshedy, Hoda G. Daabees, Sohila M. Elonsy

**Affiliations:** 1 Pharmaceutical Analytical Chemistry Department, Faculty of Pharmacy, Damanhour University, Damanhour, Egypt; 2 Pharmaceutical Chemistry Department, Faculty of Pharmacy, Damanhour University, Damanhour, Egypt

**Keywords:** Capillary Zone Electrophoresis, Greenness evaluation, Linezolid, Cefixime, Analytical Ecoscale, AGREE

## Abstract

The establishment and validation of a straightforward, accurate, and eco-friendly capillary zone electrophoretic-diode array detection (CZE-DAD) procedure has been presented for concurrent measurement of two common antibiotics, namely, linezolid (LIN) and cefixime trihydrate (CEF), in their binary mixture or combined dosage form. The selected fused silica capillary has total and effective lengths equal to 58.5 cm and 50 cm, respectively, with a 50 µm internal diameter. Injections were performed utilizing 100 mM borate buffer at pH 10.2 as the background electrolyte (BGE) with a 15.0 s injection time. The finally utilized voltage was 30 kV. DAD was programmed to measure LIN at 250 nm and CEF at 285 nm. In less than 6 min, the two cited drugs were resolved at 2.51 and 5.47 min for LIN and CEF respectively. The introduced procedure had a linear response in the concentration range of 5–50 μg/mL for both analytes with correlation coefficients > 0.9999. Detection and quantification limits were 1.213 and 4.042 μg/mL, respectively, for LIN and 0.301 and 1.004 μg/mL, respectively, for CEF. Validation was conducted according to the International Council for Harmonization (ICH), concerning linearity, detection and quantitation limits, range, accuracy, precision, selectivity, and robustness. Precision was found acceptable due to the low relative standard deviation (RSD%) values that did not exceed 1.86% either for repeatability or for intermediate precision. Additionally, the adequately recovered concentrations and the low values of percentage relative error (Er%) provide evidence of the accuracy of the proposed method. On the other hand, the robustness of the introduced method was affirmed by the acceptable RSD% values that did not exceed 0.6% after deliberate changes in the following procedure parameters: buffer concentration, buffer pH, and wavelength. Finally, the ability of the presented method to quantify the two tested drugs in laboratory-prepared tablets was confirmed by the adequate recoveries (≥ 99%) utilizing the standard-addition procedure, along with the absence of any significant difference between the proposed method and the reference method as proven by the student’s t-test and the variance-ratio F-test values that did not exceed the theoretical ones. The analytical Eco-Scale and the analytical GREEness metric (AGREE) were the tools utilized for greenness assessment. This CZE procedure is the first electro-driven separation method that was utilized for the analysis of both antibiotics in their combined laboratory-prepared tablets with no interference from the co-formulated adjuvants.

**Supplementary Information:**

The online version contains supplementary material available at 10.1186/s13065-023-01049-3.

## Introduction

In theory, the green analytical chemistry (GAC) concept is the application of sustainable principles in scientific analytical research laboratories. The green analytical chemistry (GAC) philosophy was created at the start of the twenty-first century to make the analytical processes safer for operators and more environmentally friendly [1].

Despite not being a novel idea, there was no established system for measuring the greenness of analytical chemistry. Although green chemistry metrics were available back then but they were not applicable in the assessment of different analytical methods. Therefore, more attention was received for the development of appropriate metrics for the greenness assessment of analytical chemistry procedures. Some of the developed tools are easy to use, but they don't address every aspect of how analytical processes affect the environment, while others are more inclusive but it could be challenging to use [2]. Regardless of the wide range of analytical techniques and the huge variety of the tested analytes, each analytical procedure at least involves a sample, an analyst and a minimum of one chemical reagent. Furthermore, all analytical procedures generate waste.

In the development of a green analytical procedure, there are twelve principles that represent the GAC’s key aspects. Those twelve guidelines have their abbreviations collected in the word SIGNIFICANCE. In details, (S) is to select a direct analytical procedure. The first (I) letter is to integrate the analytical process. (G) is to generate the minimum possible waste. The first (N) is to never waste energy. The second (I) letter is to implement automatic and miniaturized procedures. (F) is to favor using chemicals derived from renewable sources. The third (I) letter is to increase the operator's safety. The first (C) is to carry out measurements in situ. (A) is to avoid performing derivatization reactions. The second (N) is to note the significance of minimizing sample size and number. The second (C) is to choose a multi-parameter or multi-analyte technique. (E) is to eliminate or exchange hazardous chemicals [3, 4].

The previous 12 principles could be summarized in the following main four objectives for greening analytical methods. First is the limitation or avoidance of using different chemical substances. Second is the reduction in consuming energy. Third is the decrease in and appropriate disposal of analytical waste. Fourth is the increase in operator safety [4]. In the past years, it was common to focus on performance attributes like LOD, recovery, accuracy, and linear range while developing analytical procedures. Recently, choosing an analytical technique for testing an analyte has been based on many criteria, such as performance, green analytical chemistry values, and economic costs. It is obvious that all of these considerations must be taken into account when choosing an analytical approach. In most instances, finding a reasonable compromise between improving the reliability of the test results, and the eco-friendliness of the analytical technique is a challenge [2].

Due to the increasing concern about the human impact on the ecological system, different trials must be made to reduce the damaging environmental impact of each field of human activities. Consequently, new, greener analytical methods should be developed to replace the non-green ones. Capillary electrophoresis (CE) is considered to be a considerable substitute and supplementary technique to traditional chromatographic procedures. Numerous benefits are available, including improved resolution, quick and extremely efficient separations, small sample sizes, minimal reagent usage (aqueous buffers in nanoliter amounts), minimal energy utilization, easy instrumental set-up, and a relatively low cost per sample. Therefore, CE became an excellent, greener candidate for pharmaceutical analysis as a result of all these factors [1, 5, 6].

On the other hand, a major global issue considering the spread of multidrug-resistant (MDR) bacterial diseases is regarded as an urgent concern. The bacterial resistance to at least one antibiotic from three or more distinct classes is referred to as multidrug resistance. In light of the difficulty of treating MDR infections and their frequent high fatality rates, antibiotic combination therapy has been adopted [7]. Despite the fact that the unnecessary use of antibiotic combination therapy can aggravate the situation of antibiotic resistance, but it is mostly utilized for one or more of the following reasons: ensuring the use of a broad antibacterial spectrum, improving efficiency against infections caused by several microbes (polymicrobial infections), the synergistic effects of antibiotic mixtures over monotherapy, or the decreased chances of developing resistance to two antibiotics simultaneously as compared to one drug [7]. Considering this therapeutic trend, the development of appropriate analytical procedures for concurrent estimation of co-formulated or co-administered medications has become more crucial. The present work represents simultaneous analysis of the antibiotic combination therapy of Cefixime trihydrate (CEF) in its mixture with linezolid (LIN).

**Fig. 1:**
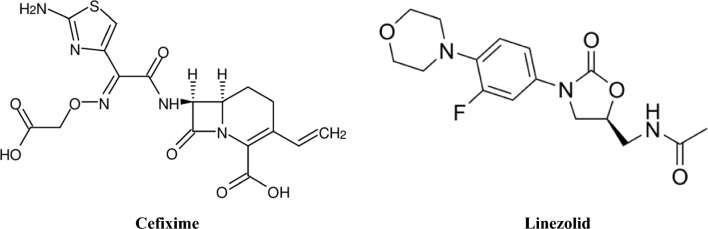
Chemical structures of the compounds investigated in the study

CEF is an antibiotic of the cephalosporins third generation. CEF chemical name is (6*R*,7*R*)-7-[[(2*Z*)-2-(2-amino-1,3-thiazol-4-yl)-2-(carboxymethoxyimino) acetyl]amino]-3-ethenyl-8-oxo-5-thia-1-azabicyclo[4.2.0]oct-2-ene-2-carboxylic acid; trihydrate (Fig. 1) [8]. Due to the fact that CEF shows high stability if beta-lactamase enzymes are present, many microorganisms that could resist penicillins and some cephalosporins may be sensitive to CEF [9].

CEF is utilized in treating different infections of the lower respiratory tract, such as bronchitis, along with gonorrhea, pharyngitis, otitis media, and urinary tract infections. CEF's mechanism of action depends on the inhibition of the production of mucopeptides in the bacterial cell wall. The third and final step of the formation of the bacterial cell wall is inhibited after the binding of CEF to a certain penicillin-binding proteins (PBPs) that are found in the bacterial cell wall. After that step, the autolytic enzymes that are present in the bacterial cell wall as autolysins start to induce cell lysis [9].

LIN is the first member of the oxazolidinone antibiotics and it’s chemical name is *N*-[[(5*S*)-3-(3-fluoro-4-morpholin-4-ylphenyl) -2-oxo-1,3-oxazolidin-5-yl] methyl] acetamide (Fig. 1) [10]. LIN is utilized in treating infections that are brought on by aerobic gram-positive bacteria, such as vancomycin-resistant *Enterococcus faecium* infections, skin infections, and nosocomial or community-acquired pneumonia. Through preventing the start of bacterial protein synthesis, LIN exhibits its antibacterial effect. It binds to a location on the 50S subunit of the 23S ribosomal RNA of bacteria and blocks the assembly of a functional 70S initiation complex, which is necessary for bacterial division and reproduction. Gram-negative infections are not advised to be treated with this medication [11].

Literature survey reveals that many analytical techniques were utilized in the analysis of CEF, such as spectrophotometry [12], High-Performance Thin Layer Chromatography (HPTLC) [13], and High-Performance Liquid Chromatography (HPLC) [14–16] in different matrices as bulk powder, pharmaceutical formulations, or biological matrices either individually or in mixture with other medications. Similarly, according to previous reports, LIN was estimated individually or in the presence of other medications in various matrices, utilizing different methods such as spectrophotometry [17–19], HPTLC [20], and HPLC [21, 22].

Gramocef L® is the marketed combined tablet formulation brand of LIN and CEF in the ratio of 600 mg: 200 mg respectively. Concurrent analysis of CEF and LIN in a fixed dose form was reported using HPLC methods [23–30], various spectrophotometric methods, including Vierodt’s method [31–35], the absorbance ratio method (Q analysis) [26, 32, 36, 37], first order derivative spectrophotometry [38], zero crossing second derivative spectrophotometry [34, 36], ratio derivative method [34, 39], ratio difference method [39] and mean centering of ratio spectra [39]. To the extent that we are aware, this is the first electro-driven separation method utilizing capillary electrophoresis for concurrent analysis of the two cited drugs. Furthermore, none of the previously published approaches were assessed for their greenness using any of the available tools.

The aim of the proposed study is to introduce a simple, smart and eco-friendly capillary zone electrophoresis method (CZE) for analysis of CEF in its binary combination with LIN simultaneously. The proposed CZE method fulfilled International Council for Harmonization (ICH) validation guidelines [40]. Furthermore, considering greenness evaluation of the presented method, two different greenness evaluation metrics, namely, analytical Eco-scale and the new analytical GREEness metric (AGREE) were utilized [3, 41].

## Experimental

### CE system

The utilized capillary electrophoresis system is based on an Agilent 7100 series CE instrument (Agilent Technologies, Waldbronn, Germany) combined with a diode array detector (DAD) and connected to a system for managing data that includes a personal computer with Agilent ChemStation Software. DAD was used for the detection of LIN at 250 nm and of CEF at 285 nm. The utilized deactivated fused silica capillary (Agilent Technologies, Waldbronn, Germany) has total and effective lengths of 58.5 cm and 50 cm, respectively, and a 50 µm internal diameter.

### Materials and reagents

CEF pure powder was a generous gift from Pharco Pharmaceuticals Company, Alexandria, Egypt, and LIN was supplied as a free sample from EVA Pharma Company, Alexandria, Egypt. Methanol of HPLC grade (Sigma-aldrich, Buchs, Switzerland) and deionized water were used as solvents. Analytical grade boric acid and sodium hydroxide were obtained from Oxford Lab Fine Chem company (Neminath Industrial Estate, Navghar, Vasai East, Palghar-410210, Maharashtra, India). Since it wasn't available on the local market, the pharmaceutical preparation for the combination of LIN and CEF was prepared in the laboratory.

### General procedure

#### Preparation of the background electrolyte

The finally utilized background electrolyte solution was borate buffer in concentration of 100 mM. Borate buffer was prepared by dissolving 0.618 g boric acid and 0.2 g sodium hydroxide in 100 mL deionized water and adjusting the pH to 10.2 with the aid of 0.1 M sodium hydroxide solution.

#### Capillary conditioning

Before the initial run, the capillary should be rinsed with 0.5 M NaOH for 15 min followed by deionized water for 15 min on every working day. After that, the equilibration process is applied by washing the capillary for 150 s with 0.1 M NaOH, waiting for another 150 s with the capillary filled with 0.1 M NaOH to guarantee that the capillary's inner wall is fully activated, flushing it for 150 s with deionized water, and finally equilibrating it for 600 s with the finally selected running buffer. Furthermore, the running buffer was used to flush the capillary for 60 s between each pair of subsequent injections. Refilling both buffer vials after every six consecutive runs is important to sustain the repeatability of run-to-run injections. The hydrodynamic injection technique was performed for 15 s using 50 mbar pressure. The applied voltage during each run is equal to 30 kV.

#### Preparation of standard solutions and construction of calibration graphs

Standard stock solutions were separately prepared in methanol of HPLC grade to contain 1000 μg/mL of LIN and CEF and kept at + 4 °C. To achieve concentrations ranging from 5 to 50 μg /mL for the tested medicines, different portions of both stock solutions were diluted to 10 mL with deionized water in a set of 10 mL calibrated flasks. Triplicate injections were carried out for each concentration. Finally, calibration graphs were created by plotting peak areas against the equivalent concentrations.

### Methods validation

Validation of the introduced CZE method was conducted in compliance with ICH regulations concerning linearity, detection and quantitation limits, range, accuracy, precision, selectivity, and robustness [40].

#### Linearity and concentration ranges

The linearity of the introduced approach was evaluated by analyzing different serially diluted concentrations of both medicines between 5 and 50 µg/ml.

#### Detection and quantification limits

The concentration of an analyte that has a signal: noise ratio = 3:1 is considered the limit of detection (LOD). On the other hand, the concentration of an analyte that has a signal: noise ratio = 10:1 is considered as the limit of quantitation (LOQ). For the determination of both values five replicates of the lowest concentration in the linear range (5 µg/ml) were analysed. The signal to noise ratios for those replicates was obtained utilizing the Agilent ChemStation Software and then the average signal to noise ratio was calculated. The finally calculated signal to noise ratio was utilized to calculate both LOD and LOQ.

#### Accuracy and precision

The intra-day (repeatability) and inter-day (intermediate precision) of the suggested procedure were evaluated by calculating the corresponding responses of three concentration levels within the specified linearity range, three times on the same day and three times on three different days, respectively.

#### Selectivity and analysis of the laboratory prepared mixtures

The proposed CZE method was applied for the analysis of four laboratory-made mixtures of both tested analytes in their specified linear ranges in various concentrations in order to study selectivity. These mixtures resemble different ratios, equal to or around those present in the fixed dose combination as presented in Table 4.

#### Robustness

The reliability of an analytical technique and its ability to be unaffected by deliberate changes in the procedure parameters are evaluated by the robustness test of the analytical procedure. The robustness of the developed procedure was confirmed by testing both compounds in a mixture of 30 μg/mL each. The evaluated variables were buffer concentration (100 ± 2 mM), buffer pH (10.2 ± 0.2), and wavelength (± 2 nm) which were deliberately and separately varied. Triplicate injections were carried out, with only one alteration was performed each time.

#### Stability of solutions

Stability was assessed by storing the finally prepared sample solutions and standard working solutions for 24 h at ambient temperature or for one week at 4 °C, along with the stock solutions after a week of refrigeration at 4 °C. Finally, triplicate injections were carried out from each solution.

### Assay of the laboratory prepared tablets

As a result of the lack of a combined CEF/LIN dosage form in the Egyptian market, the introduced CZE method was utilized for concurrent analysis of the tested antibiotics in their laboratory-prepared tablets. To imitate the ratio of both medicines in the brand Gramocef L®, ten laboratory-made tablets were prepared, each containing 600 mg LIN and 200 mg CEF. Along with different tablet excipients, which are magnesium stearate, flour, aerosil, and lactose, the quantity of LIN and CEF was measured, powdered, and homogeneously mixed. In the presented work for the preparation of the laboratory-made tablets, the exact excipients utilized in the commercially combined tablets for the tested drugs couldn’t be assumed. On the other hand, excipients that are used in the manufacturing of single component products containing either linezolid or cefixime alone were found. The linezolid tablet brand Linezolid® states that it contains only lactose as an excipient [42]. The cefixime tablet brand Gramocef-O-200® states that it contains magnesium stearate, aerosil, talc, sodium lauryl sulphate, and dibasic calcium phosphate as excipients [43]. In the presented work, only magnesium stearate, flour, aerosil, and lactose were used as excipients so as not to make laboratory-prepared tablet with an unacceptably high weight. Additionally, the nonutilized excipients as talc, sodium lauryl sulphate, and dibasic calcium phosphate would not be able to interfere with the analysis steps as those excipients are not soluble in methanol, which is the solvent used for tablet extraction.

An accurate weight of the produced tablets, comprising 150 mg LIN and 50 mg CEF, was mixed with 15 mL of HPLC grade methanol, vortexed for 5 min, and finally filtered in a 50 mL calibrated flask. The remaining residue was rinsed twice with 3 mL of methanol, and washings were collected with the filtrate. The final volume was completed to 50 mL with methanol to obtain a sample extract stock solution of 3 mg/ml LIN and 1 mg/ml CEF. To get final concentrations within the desired linear ranges, portions of the final tablet extraction solution were diluted with deionized water. The prepared dilutions were then handled as directed by "General Procedure". Recovery values were calculated using regression data.

For standard addition assay, portions of LIN and CEF stock solutions were added to portions of the extracted sample solution, to attain final concentrations in the specified linear ranges. After this, the assay was conducted as described in the "General Procedure". Recovery values were derived by contrasting the response of each analyte with the increment response discovered after adding the required standard.

## Results and discussion

### Method optimization

Numerous factors influence the use of capillary zone electrophoresis in the separation of analytes. Therefore, various trials were performed to determine the optimum experimental parameters such as buffer (type, pH, and concentration), voltage, diluting solvent, injection (technique and time), and detection wavelengths in order to finally achieve symmetrical peaks with as short migration times as possible.

#### Buffer type and pH selection

In the separation of analytes using capillary electrophoresis, the pH of the background electrolyte (BGE) plays an essential role, particularly for analytes with weak acidic or basic characteristics. LIN owes an acidic pKa value = 14.85 and a basic pKa value of − 1.2 [11]. Those pKa values indicate that LIN couldn’t be ionized at the pH range of 3–11 that is usable in capillary electrophoresis applications. Due to its two carboxyl groups, CEF has an acidic nature, with a pK_a_ value of 3.54, which indicates that CEF primarily exists in the negatively ionized state at any pH higher than 3.54 by two pH units [44]. Several trials were conducted to find the optimal buffer pH for LIN and CEF separation.

Utilizing a fused silica capillary of 50 µm internal diameter and 30 cm effective length, 25 mM concentrations of acetate buffer at pH = 4.6, phosphate buffer at pH = 7.4, and borate buffer at pH = 9.2 were tried individually as BGE. Acetate buffer showed distorted and fronted peaks, especially for CEF, as predicted from its pKa, as CEF is not completely ionized at 4.6 pH (Additional file 1: Fig. S1). On the other hand, 25 mM borate buffer at pH = 9.2 showed an enhanced peak shape with a smoother baseline than 25 mM phosphate buffer at pH = 7.4, but unfortunately, it led to separation of both drugs in less than 1.6 min, consequently the capacity (retention) factor K’ had an unacceptable value of less than 2 for each compound (Additional file 1: Fig. S1). Subsequently, utilizing taller capillary of 50 cm effective length showed longer migration times with acceptable capacity factor values. No trials were performed on capillaries taller than 50 cm, as that would lead to unnecessarily longer migration times. Further pH studies were performed on borate buffer at different pH values (from 8 to 11); those trials showed no effect on the separation of the two tested compounds, but it was revealed that a pH of 10.2 gave the optimal peak shape and width. In the present work, the finally selected optimum background electrolyte is borate buffer in a concentration of 100 mM with a pH of 10.2. Regarding the pKa values of the two tested drugs, LIN should be neutral and CEF should carry a negative charge in the selected buffer pH.

Considering the basic principle of conventional capillary electrophoresis, the injection is performed at the anode and the detection is conducted at the cathode. Regarding CZE, after injecting the sample in a capillary filled with buffer, analytes will migrate as zones through the capillary after the application of the voltage. The migration rate of each analyte depends on its electrophoretic mobilities. As the direction of the EOF’s movement is from the anode to cathode, so the tested compounds will elute in the following order: cations, then neutral compounds, followed by anions under the effect of both electrophoretic mobility and electroosmotic flow [45]. According to that, the migration order of the tested analytes could be easily explained; LIN migrates first out of the capillary as it is found in a neutral form in the BGE, then CEF as it has a negative charge.

#### Buffer concentration

In the CZE technique, the concentration of the BGE plays an important role in the separation of analytes. The impact of buffer concentration was tested using different concentrations of borate buffer at pH 10.2, ranging from 10 to 100 mM, with applying 30 kV. Varying buffer concentrations from 10 to 60 mM did not affect migration times significantly. On the other hand, buffer concentrations from 60 to 100 mM showed an increase in migration times, especially for the CEF peak, as shown in Additional file 1: Fig. S2. On the other hand, as the separation and resolution between the two peaks were fulfilled utilizing all buffer concentrations, peak shape was put into consideration in order to choose the optimum buffer concentration. The improvement in peak shape utilizing higher buffer concentration was a result of the high-field stacking of the analyte generated by the difference between the low and high ionic strengths of the diluent (water) and BGE, respectively. This difference forces ions to migrate more quickly and to stack in a sharper peak [46]. 100 mM borate buffer showed better peak height, width, and symmetry values compared to those obtained with lower buffer concentrations, especially for the LIN peak (Additional file 1: Table S1).

#### Applied voltage

Using the finally selected BGE, several voltage values (10, 15, 20, 25, and 30 kV) were evaluated. It was clearly noticed that, when applied voltage decreased, migration times elongated owing to the reduction of the electroosmotic flow (EOF). In the presented work resolution wasn't the main factor in selecting the optimum voltage, as it was acceptable at all tested voltages. On the other hand, as voltage dropped, peaks widened and peak symmetry changed. Therefore, a voltage of 30 kV was determined to be appropriate, as it permitted separation with the least migration times and the best peak symmetry, as presented in Additional file 1: Fig. S3.

#### Sample diluting solvent selection

Water and background electrolyte (BGE) were compared for usage as a diluent. Although there was no difference between both of them in migration times and peak areas. On the other hand, peak shape was improved by using water as a diluent. This improvement is a result of high-field stacking of the analyte.

#### Sample injection time

Peak width and height are affected by injection time in the hydrodynamic injection technique. Using the final optimal conditions, injection times and peak heights are directly related. A sample was injected hydrodynamically at a pressure of 50 mbar, and the injection time was changed between 3 and 15 s. Although, injection time and peak height are directly correlated, a further increase in injection times also resulted in linearity deviation and peak shape distortion. Therefore, to maintain that linear relation and to ensure optimum peak symmetry, the utilized injection time was 15 s.

#### Wavelength for detection

**Fig. 2:**
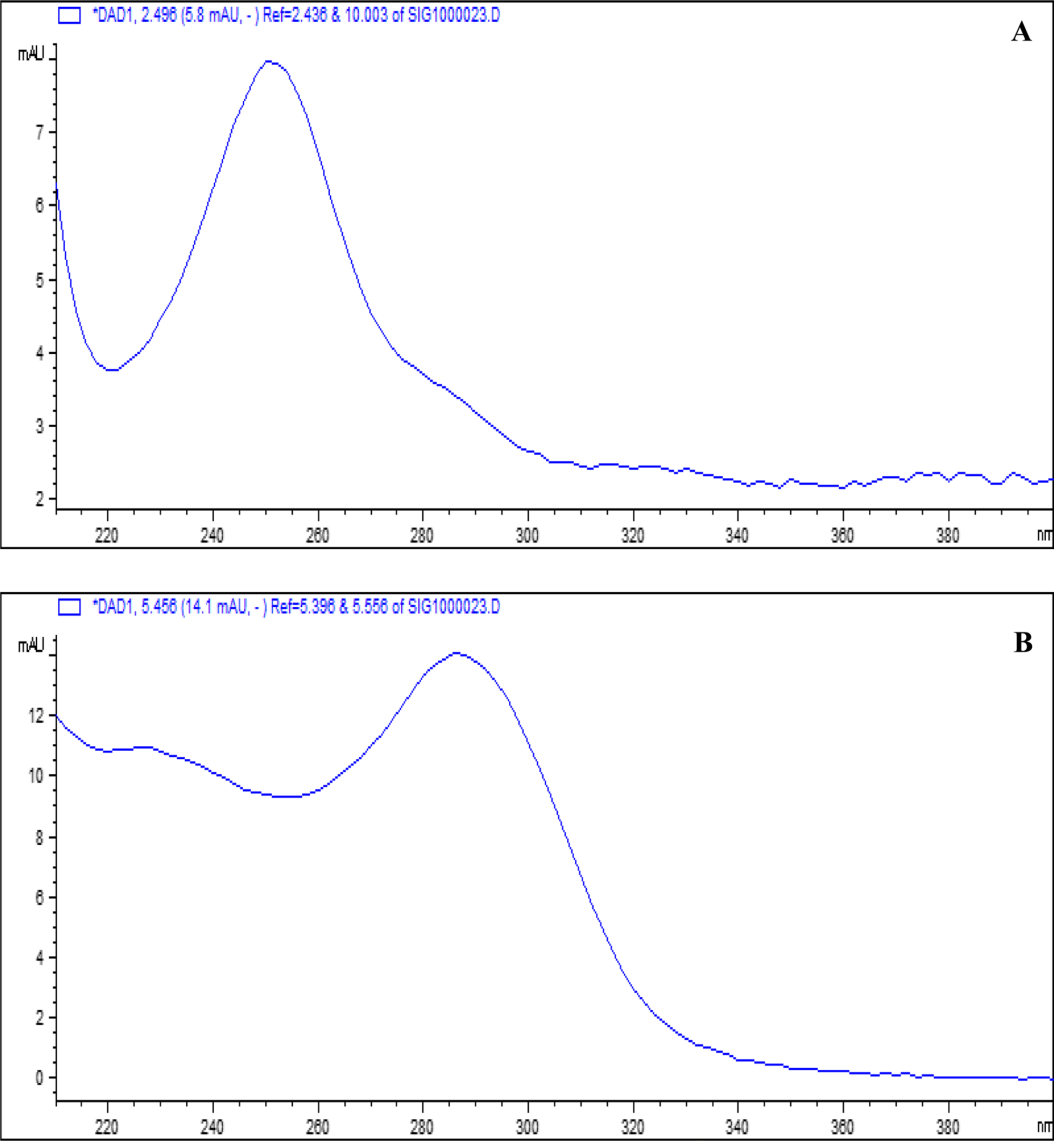
UV spectrum of (**A**) Linezolid (LIN) and (**B**) Cefixime (CEF)

Among the benefits of utilizing a diode array detector is that it can measure analytes at various wavelengths. In this way, each substance could be detected using its maximum wavelength of absorption, which enhances the sensitivity of the presented method. Figure 2 demonstrates the ultraviolet spectra of both drugs in the finally optimized running buffer. LIN and CEF were examined at 250 and 285 nm, respectively.

**Fig. 3:**
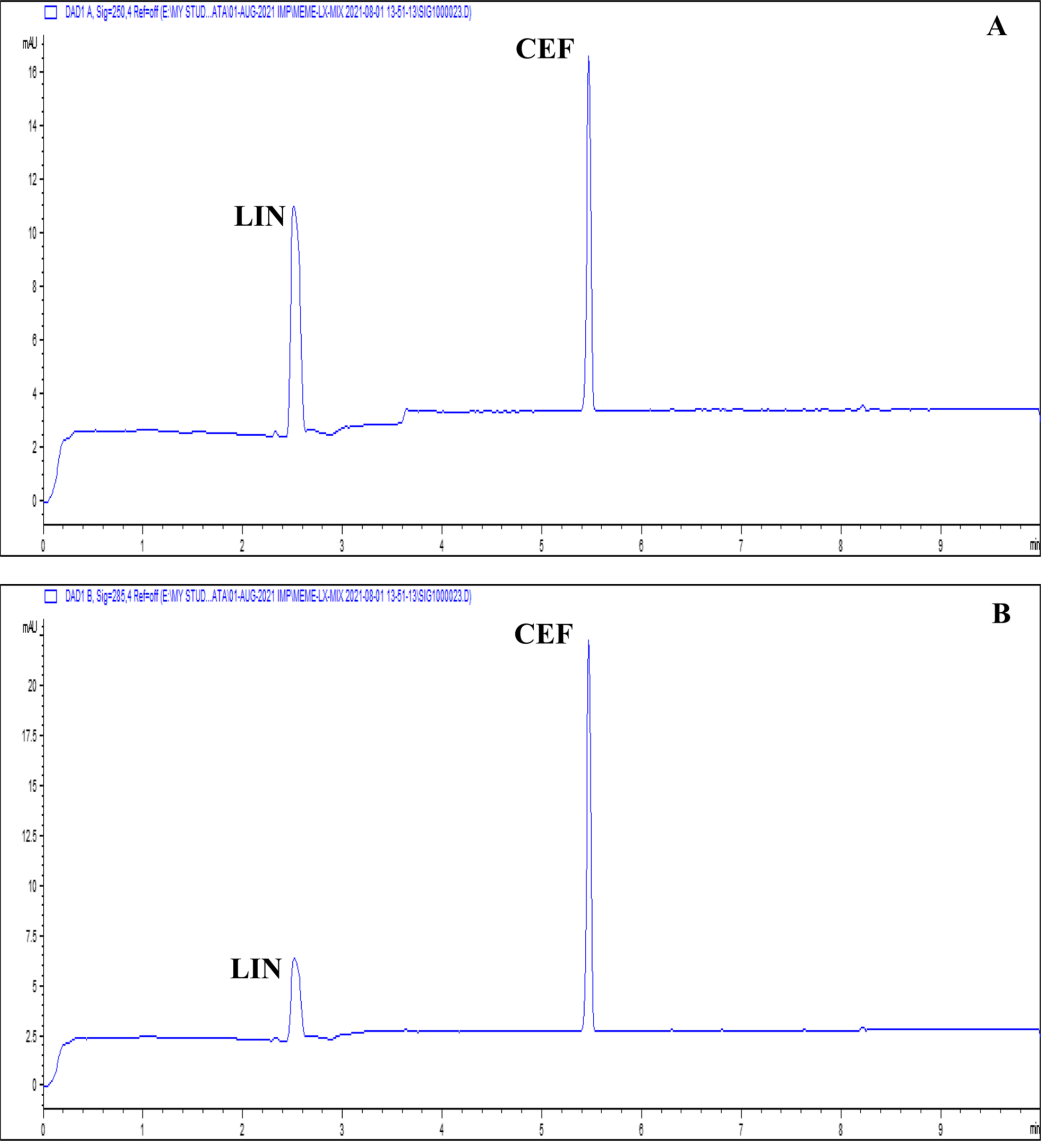
CZE electropherograms of a standard mixture containing 45 µg/mL of both LIN and CEF at (**A**) 250 nm for LIN and at (**B**) 285 nm for CEF

The summary of the optimization procedure shows that the finally proposed method utilized a fused silica capillary of internal diameter, total length, and effective length equal to 50 μm, 58.5 cm, and 50 cm, respectively. Runs were performed using BGE of 100 mM borate at pH = 10.2, 15.0 s injection time, and an applied voltage of 30 kV. DAD was operated to measure LIN at 250 nm, and at 285 nm for CEF. The presented method enabled separation of the two tested compounds in less than 6 min, according to the CZE electropherogram (Fig. 3). Migration times were 2.51 and 5.47 min for LIN and CEF, respectively (Table 1). System suitability parameters reveal good resolution between both peaks that was achieved by the proposed method. Furthermore, additional system suitability parameters were evaluated and confirmed to be satisfactory (Table 1).

**Table 1:** System suitability parameters for CZE-DAD analysis of LIN and CEF mixture

Parameter	LIN	CEF
t_R_ ± SD (min) Retention factors (k') Theoretical plates (N) USP tailing factor Selectivity (ɑ) Resolution (R_s_)	2.51 ± 0.02 4.02 3092 1.30 – –	5.47 ± 0.05 9.94 72,222 0.97 2.47 22.44

### Method validation

#### Linearity and concentration ranges

Peak areas and concentrations of LIN and CEF are linearly correlated between 5 and 50 µg/ml (Table 2). Different validation items, such as correlation coefficients (r), intercepts, slopes with their standard deviations, and standard deviations of residuals (S_y/x_) are summarized in Table 2. The high (r) values confirmed the linearity of the calibration plots (where r = 0.9999 for both CEF and LIN). Acceptable linearities were also proved by high F values, along with negligible intercepts and small significance F for both drugs. Furthermore, the RSD% of the slope was less than 1% for each analyte, which indicates good linearity.

**Table 2:** Analytical parameters for determination of LIN and CEF mixture using the proposed CZE-DAD method

Parameter	LIN	CEF
Wavelength (nm)	250	285
Concentration range (μg/mL)	5–50	5–50
Intercept (a)	17.971	1.583
S_a_^a^	0.146	0.275
Slope (b)	1.098	1.381
S_b_^b^	0.005	0.009
RSD% of the slope (S_b_%)	0.455	0.652
Correlation coefficient (r)	0.9999	0.9999
S_y/x_^c^	0.205	0.353
F^d^	46,730	23,223
Significance F	2.04 × 10^–11^	1.11 × 10^–8^
LOD^e^ (μg/mL)	1.213	0.301
LOQ^f^ (μg/mL)	4.042	1.004

^a^Standard deviation of the intercept

^b^Standard deviation of the slope

^c^Standard deviation of residuals

^d^Variance ratio, equals the mean of squares due to regression divided by the mean of squares about regression (due to residuals)

^e^Limit of detection

^f^Limit of quantification

#### Detection and quantification limits

The LOD and LOQ values for CEF, and LIN were listed in Table 2. The introduced CZE method's sensitivity is approved by the LOD and LOQ values.

#### Accuracy and precision

The intra-day and inter-day replicates revealed that RSD% values were less than 2% in the analysis of either LIN or CEF (Table 3). Therefore, the repeatability and intermediate precision of the proposed method were found acceptable due to these low RSD% values. Concentrations that have been adequately recovered and low values of percentage relative error (Er%) provide additional evidence of the accuracy of the proposed method.

**Table 3:** Precision and accuracy for determination of LIN and CEF in bulk form using the proposed CZE-DAD method

Analyte	Nominal value (μg/ml)	Found ± SD^a^ (μg/ml)	RSD (%) ^b^	E_r_ (%) ^c^
LIN	*Within-day*			
	10	10.06 ± 0.08	0.80	0.60
	30	29.96 ± 0.52	1.74	0.13
	45	45.13 ± 0.14	0.31	0.29
	*Between-days*			
	10	10.03 ± 0.12	1.20	0.30
	30	29.99 ± 0.18	0.60	0.03
	45	44.78 ± 0.19	0.42	0.49
CEF	*Within-day*			
	10	10.11 ± 0.14	1.38	1.10
	30	29.52 ± 0.55	1.86	1.60
	45	45.45 ± 0.60	1.32	1.00
	*Between-days*			
	10	10.11 ± 0.14	1.38	1.10
	30	29.83 ± 0.15	0.50	0.57
	45	45.09 ± 0.36	0.80	0.20

^a^Mean ± standard deviation for three determinations

^b^% Relative standard deviation

^c^% Relative error

#### Selectivity

**Fig. 4:**
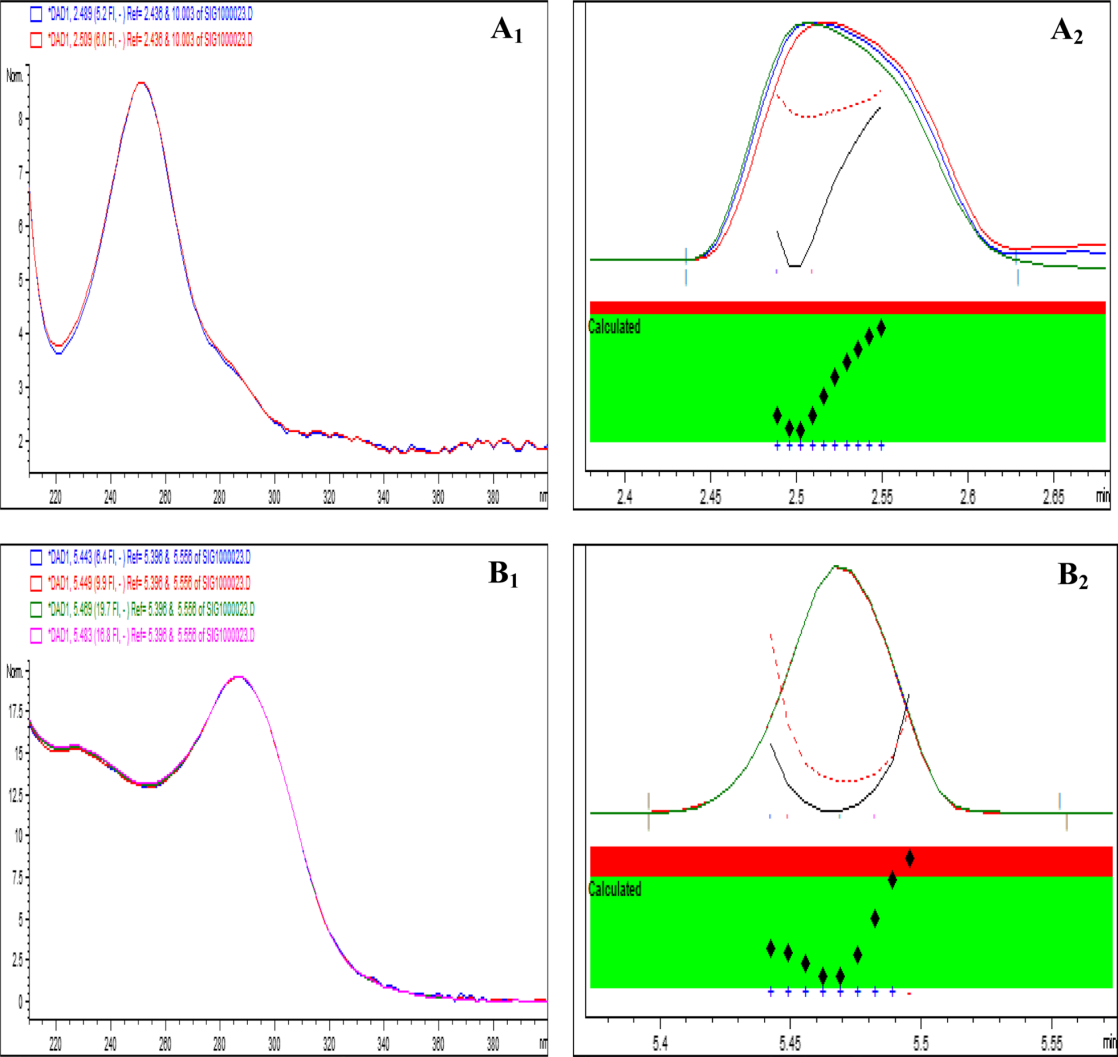
Absorption spectrum of (**A**_**1**_) LIN and (**B**_**1**_) CEF measured at different time intervals across the peak. While (**A**_**2**_) and (**B**_**2**_) represents the purity plot for LIN and CEF peaks respectively

The analysis's findings for testing laboratory-made mixtures, which included recovery values, RSD%, and Er%, confirmed the developed method's selectivity and showed its capacity to separate and quantify the tested analytes in various ratios (Table 4). Furthermore, there was no indication of any co-eluted peak from the inactive ingredients, as proved by the electropherogram of a blank sample after extraction of a placebo laboratory-prepared tablet that contains only the excipients without the two tested drugs (Additional file 1: Fig. S4). Additionally, peak purity and selectivity were confirmed using DAD, which allows peak purity verification, by the superimposition of a collection of UV spectra at various points across each peak (Fig. 4 (A_1_ and B_1_)). Peak's similarity factor increases the sensitivity and trustworthiness of peak purity assessment, might be derived by taking into account all of the spectra obtained within the elution of a peak, not only 3 or 4 spectra. As the noise threshold value was not exceeded by the similarity curve, as seen by the red area in Fig. 4, each peak had a high degree of similarity.

**Table 4:** Determination of LIN and CEF in laboratory-prepared mixtures using the proposed CZE-DAD method

Nominal value (μg/ml)	Found ± SD^a^ (μg/ml)	RSD (%) ^b^	E_r_ (%) ^c^
	LIN		
Mixture 1 (LIN: CEF = 15:15)	15.15 ± 0.20	1.32	1.00
	15.25 ± 0.20	1.31	1.67
Mixture 2 (LIN: CEF = 15:5)	29.58 ± 0.14	0.47	1.40
	44.88 ± 0.11	0.25	0.27
	CEF		
Mixture 3 (LIN: CEF = 30:10)	15.06 ± 0.06	0.40	0.40
	5.06 ± 0.06	1.19	1.20
Mixture 4 (LIN: CEF = 45:15)	10.01 ± 0.07	0.70	0.10
	15.17 ± 0.25	1.65	1.13

^a^Mean ± standard deviation for five determinations

^b^% Relative standard deviation

^c^% Relative error

#### Robustness

The robustness of the introduced method was assessed after calculating the SD and RSD% for peak areas and migration times after a few planned adjustments to the experimental conditions. The variations in responses of LIN and CEF were recorded, and the assay values were estimated along with each varied parameter (Table 5). The investigated changes did not considerably influence either peak area or migration times of the analysed medicines, as confirmed by RSD% values, demonstrating the robustness of the approach (Table 5).

**Table 5:** Robustness evaluation for the analysis of LIN and CEF mixture using the proposed CZE-DAD method

Parameter	LIN*			
	Peak area ± SD	RSD%	Migration time ± SD	RSD%
Buffer concentration 100 ± 2 m mol	56.70 ± 0.10	0.18	2.53 ± 0.02	0.79
Buffer pH 10.2 ± 0.2 pH unit	57.57 ± 0.15	0.26	2.48 ± 0.03	1.21
Wavelength 250 ± 2 nm	57.17 ± 0.21	0.37		

Parameter	CEF*			
	Peak area ± SD	RSD%	Migration time ± SD	RSD%
Buffer concentration 100 ± 2 m mol	42.91 ± 0.25	0.58	5.50 ± 0.05	0.91
Buffer pH 10.2 ± 0.2 pH unit	43.15 ± 0.17	0.39	5.44 ± 0.03	0.55
Wavelength 285 ± 2 nm	43.45 ± 0.23	0.53		

^*^ Robustness parameters were determined for a mixture containing 30 µg/mL of each LIN and CEF

#### Stability of solutions

It was confirmed that sample solutions and standard working solutions were stable in water after storing them for 24 h at ambient temperature or for one week at 4 °C. Additionally, the stock solutions remained stable after at least a week of refrigeration at 4°C. Analysis showed that both tested medications were stable in these conditions, as proven by the absence of any chromatographic alterations. Furthermore, migration times, peak area values, and peak purity showed no significant changes.

### Application of the validated method in the assay of the laboratory prepared tablet

**Fig. 5:**
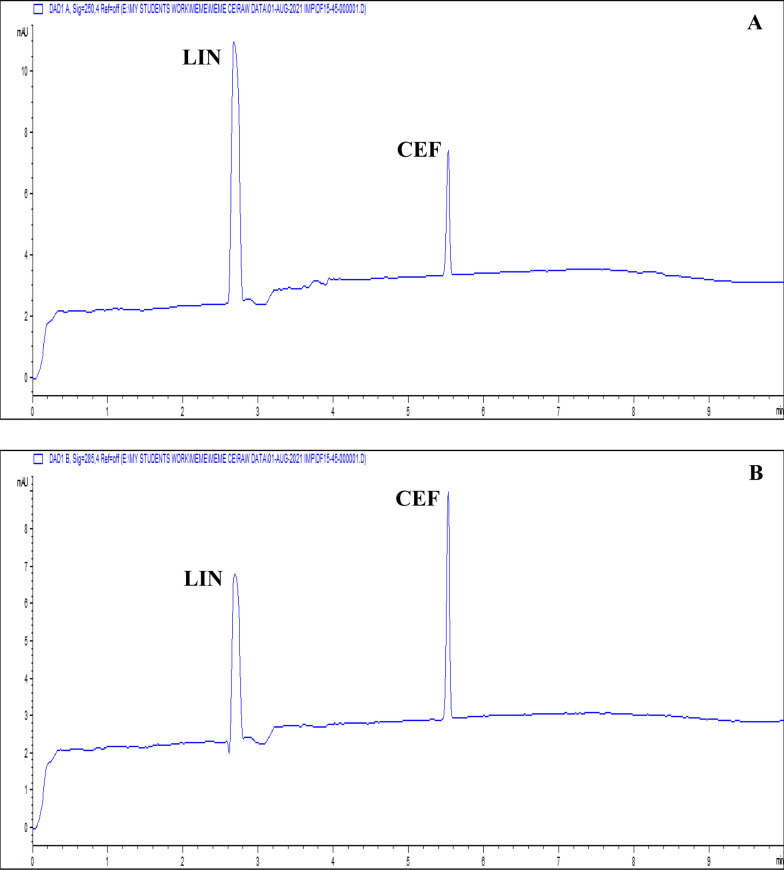
CZE electropherograms of a sample mixture obtained from the laboratory prepared LIN and CEF tablets of 45 μg/mL LIN and 15 μg/mL CEF at (**A**) 250 nm for LIN and at (**B**) 285 nm for CEF

The assay of LIN and CEF in laboratory-prepared tablets was performed using the validated CZE method. Sample preparation was performed as described under "Assay of laboratory-prepared tablet" and recoveries were measured utilizing both standard addition and external standard techniques. Figure 5 displays representative electropherograms derived from the extract of the laboratory made tablets. Both tested substances eluted at their appropriate migration times. Additionally, DAD permits peak purity confirmation as there was no indication of co-elution from any inactive component. According to the assay findings' % recovery, SD, and RSD% values, the accuracy and precision were satisfactory (Table 6). The results of the proposed method were statistically compared to those of the ratio difference spectrophotometric reference method [39] using the student *t*-test and variance ratio *F*-test. The statistical values did not exceed the theoretical ones of the 95% confidence level verifying the absence of any significant difference between the compared methods (Table 6). The presented results proved the ability of the presented procedure for routine analysis of LIN and CEF in their fixed dose combinations, requiring only few steps for sample preparation with acceptable selectivity, accuracy, and precision.

**Table 6:** Application of the proposed CZE-DAD method for analysis of LIN and CEF mixture in pharmaceutical tablets

Laboratory prepared tablets	External Standard		Reference method [39]		Standard Addition	
	LIN	CEF	LIN	CEF	LIN	CEF
%Recovery ± SD^a^	99.84 ± 0.59	99.55 ± 0.94	100.17 ± 0.46	99.59 ± 1.17	100.10 ± 0.54	99.96 ± 0.31
RSD%^b^	0.59	0.94	0.46	1.17	0.54	0.31
*t*	1.00	0.05				
*F*	1.67	1.52				

^a^Mean ± standard deviation for five determinations

^b^% Relative standard deviation

Quantification was carried out at the following wavelengths: 250 nm for LIN and 285 nm for CEF

Theoretical values for *t* and *F* at P = 0.05 are 2.31 and 6.39, respectively

### Assessment of greenness of the proposed methods

To limit their harmful impact on the environment and their potential health risks, it is critical to evaluate the proposed analytical methods in light of green analytical chemistry procedures. The application of more than one flexible green analytical chemistry metric for evaluation of the greenness of the analytical procedure is critical to assure that the suggested method is environmentally friendly by gathering as much valuable information as possible [1].

In our presented work, the approved CZE method was verified concerning greenness using two methods: the analytical Eco-Scale and the new Analytical Greenness metric (AGREE) [3, 41]. Considering the previously reported methods based on what we know for the analysis of LIN and CEF, they showed only the use of spectrophotometric and HPLC chromatographic techniques. Therefore, the newly developed CZE method was compared in term of greenness with three of the previously reported methods: two spectrophotometric articles [34, 39], one of them is a previous work of our research group [39], and a HPLC–UV [30] method as a representative example.

The first greenness assessment tool is the analytical Eco-Scale method. The analytical Eco-Scale has the benefit of being both semi-quantitative and easily calculated. This simple tool evaluates each analytical method in four primary aspects which are the consumed chemicals, the energy utilization, the occupational hazards, and finally the produced waste. According to this approach, the green ideal analytical method has a 100-point total with no penalty points (PP). The PPs are established and deducted from 100 for every deviation from the ideal status. Final scores higher than 75 prove excellent greenness; scores between 50 and 75 describe reasonable greenness; and scores below 50 represent insufficient greenness [41]. Table 7 presents the calculated analytical Eco-Scale scores for the introduced CZE method and the three selected reported procedures. In term of scores, the three compared methods showed excellent greenness. However, the proposed CZE method came in first with a score of 98, indicating that it is the greenest method, followed by both reported spectrophotometric methods and then the reported HPLC method.

**Table 7:** Penalty points of the proposed CZE-DAD method according to the Analytical Eco-scale in comparison with three previously reported methods

Evaluation Points	The proposed CZE methods	The reported 1st Spectrophotometric method [39]	The reported 2nd Spectrophotometric method [34]	The reported HPLC method [30]
1—Chemicals				
Water	0	0	–	0
Borate buffer	1	–	–	–
Methanol	–	–	6	6
2—Energy	0	0	0	1
3—Occupational hazards	0	0	0	0
4—Waste	1	3	3	3
PPs	2	3	9	10
Eco-scale score	98	97	91	90

The second greenness assessment tool is the AGREE metric, which is considered to be the most modern and user-friendly automated tool for evaluating greenness. It is utilized through a free, simple calculator built on the twelve concepts of green analytical chemistry. The resulting pictogram is divided into twelve sectors, with the finally calculated score placed in the middle, which is a score from zero to one. The level of adherence to GAC principles is represented by a specific colour assigned to each area. A dark green colour in all pictograms and a score equals 1 are the descriptions of the ideal green method according to AGREE metric. In our comparison, the proposed CZE method showed the perfect greenness with a score of 0.94, followed by the 1^st^ reported spectrophotometric method [39] with a score of 0.89, and the 2nd reported spectrophotometric method [34] with a score of 0.78, and finally the HPLC method with a score of 0.71, as shown in Table 8. The resultant greenness assessment proved the superiority of the capillary electrophoresis method.

**Table 8:** Greenness evaluation of the proposed CZE-DAD method together with three previously reported methods using AGREE Metrix

Methods and chromatographic conditions	AGREE
The proposed CZE method	
The reported 1st Spectrophotometric method [39]	
The reported 2nd Spectrophotometric method [34]	
The reported HPLC method [30]	

As CE is able to separate and analyze several analytes utilizing a few nanoliter injection volumes. CE is also performed basically in aqueous solutions, with no need to consume large volumes of different organic solvents [5].

### Comparison of the proposed methods and the previously reported methods

Literature review revealed that there is no previously published method for analyzing the selected combination using capillary electrophoresis. The presented method was compared in term of some points regarding the analytical performance with three of the previously reported methods, two spectrophotometric articles [34, 39], and a HPLC–UV [30] reported method as a representative example. It was found that the introduced approach was of comparable or slightly lower sensitivity, as proved by the linearity range, LOD, and LOQ values that are presented in Additional file 1: Table S2. Regarding sample volume and waste volume per one analysis, the introduced method revealed extensively low volumes in nanoliters as calculated utilizing the recent CEToolbox application [47]. Consequently, the introduced method has comparable sensitivity that is sufficient to the scope of analysis, along with negligible injection and waste volumes. This balance between the analytical performance and the greenness of any developed analytical method is the main idea behind the sustainability concept that is currently being promoted on a global scale [48].

## Conclusion

The introduced method represents the first use of CZE technique for estimation of LIN and CEF. Being an efficient separation technique, the CZE allowed for simultaneous estimation of the two analytes in a short runtime of less than 6 min. Moreover, regarding various validation requirements, the proposed CZE technique displayed excellent analytical performance. Additionally, it provides a fast, easy, affordable, and accurate method for identifying the two medicines in their pharmaceutical preparations. The successfully developed analytical method was evaluated, utilizing two metrics for greenness assessment the analytical Eco-Scale and AGREE. Our approach demonstrates that CE can be used as a substitute for HPLC. The benefits of CZE include low cost, flexibility, and ease of use (requiring only inexpensive capillaries, a few millilitres of running buffer, and a short run time), and it can support green chemistry by using small amounts of organic solvents only during preparation steps and avoiding hazardous solvents. The suggestion of CZE approaches for use in pharmaceutical quality control units was supported by all of the previously mentioned evaluation and validation tests.

### Supplementary Information

**Additional file 1: Figure S1.** CZE electropherograms of the separation of a standard mixture containing 50 µg/mL of both LIN and CEF using fused silica capillary of (50 µm internal diameter and 30 cm effective length), by (A) 25 mM acetate buffer at pH = 4.6, (B) 25 mM phosphate buffer at pH = 7.4, and (C) 25 mM borate buffer at pH = 9.2. **Figure S2.** Effect of buffer concentration on migration times of the two tested drugs. **Figure S3.** Effect of applied voltage on migration times of the three tested drugs. **Figure S4.** CZE electropherogram of a blank sample after extraction of a placebo laboratory prepared tablet that contains only the excipents without the two tested drugs. **Table S1.** Effect of buffer concentration on peak hight, width, and symmetry. **Table S2.** Comparison between the proposed CZE-DAD method with three previously reported methods regarding analytical performance.

## Data Availability

All data generated or analyzed during this study are included in this published article.

## Abbreviations

AGREE: Analytical GREEness metric tools
BGE: Background electrolytes
CE: Capillary electrophoresis
CZE: Capillary zone electrophoresis
CEF: Cefixime trihydrate
DAD: Diode array detector
EOF: Electroosmotic flow
GAC: Green analytical chemistry
ICH: International Council for Harmonization
LIN: Linezolid
LOD: Limit of detection
LOQ: Limit of quantitation
RSD%: Relative standard deviation
Er%: Percentage relative error
