# Supplementary material for: Development, validation and greenness assessment of a new electro-driven separation method for simultaneous analysis of cefixime trihydrate and linezolid in their fixed dose combination
Source: BMC Chem. 2023 Oct 4;17(1):132. doi: 10.1186/s13065-023-01049-3 (PMC10548557; doi:10.1186/s13065-023-01049-3)
Supplement: Supplementary file 1 — Additional file 1: Figure S1. CZE electropherograms of the separation of a standard mixture containing 50 µg/mL of both LIN and CEF using fused silica capillary of (50 µm internal diameter and 30 cm effective length), by (A) 25 mM acetate buffer at pH = 4.6, (B) 25 mM phosphate buffer at pH = 7.4, and (C) 25 mM borate buffer at pH = 9.2. Figure S2. Effect of buffer concentration on migration times of the two tested drugs. Figure S3. Effect of applied voltage on migration times of the three tested drugs. Figure S4. CZE electropherogram of a blank sample after extraction of a placebo laboratory prepared tablet that contains only the excipents without the two tested drugs. Table S1. Effect of buffer concentration on peak hight, width, and symmetry. Table S2. Comparison between the proposed CZE-DAD method with three previously reported methods regarding analytical performance. [file 13065_2023_1049_MOESM1_ESM.docx]

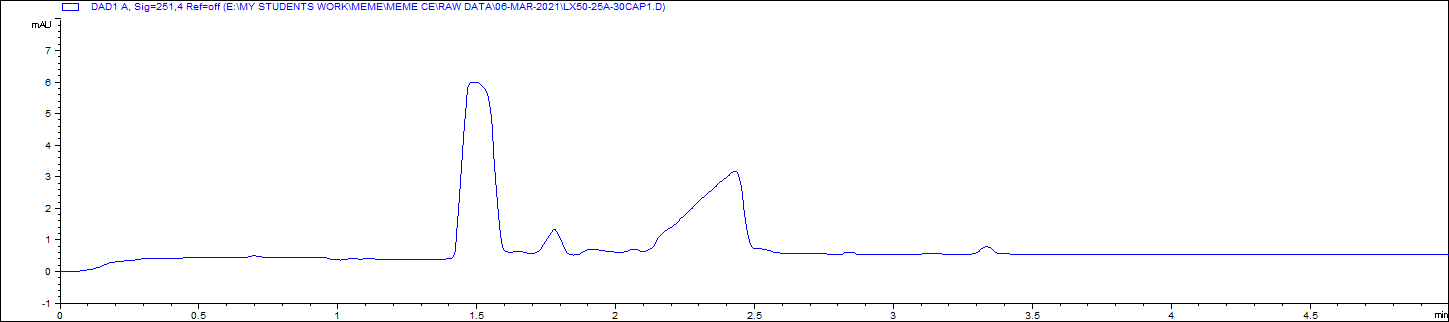


**A**

**CEF**

**LIN**


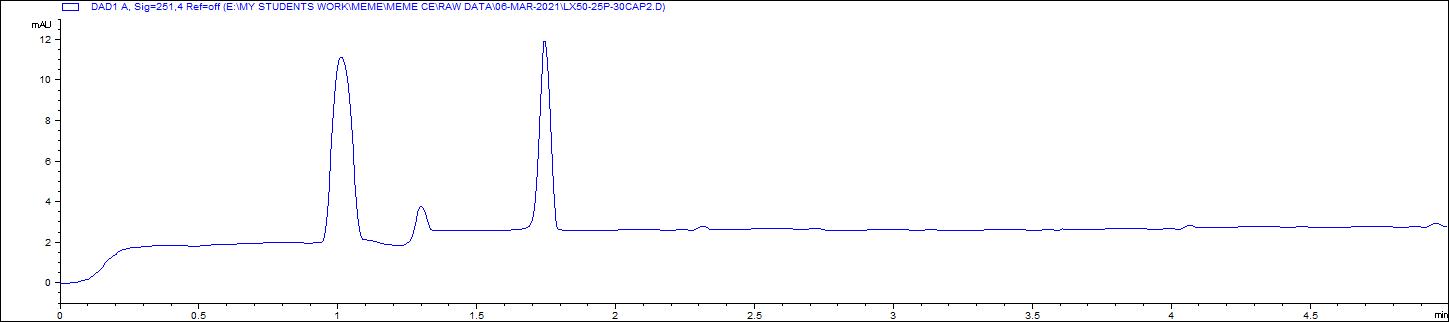


**B**

**CEF**

**LIN**


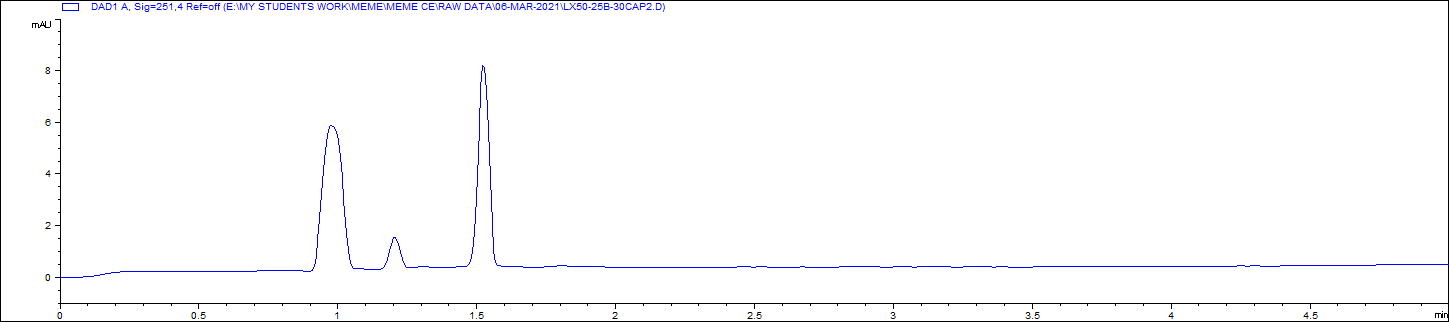


**C**

**CEF**

**LIN**

**Figure S1 : CZE electropherograms of the separation of a standard mixture containing 50 µg/mL of both LIN and CEF using fused silica capillary of (50 µm internal diameter and 30 cm effective length), by (A) 25 mM acetate buffer at pH = 4.6, (B) 25 mM phosphate buffer at pH = 7.4, and (C) 25 mM borate buffer at pH = 9.2.**


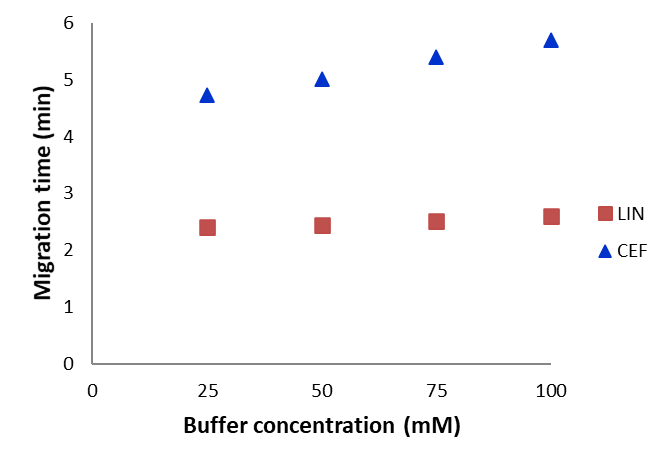


**Figure S2: Effect of buffer concentration on migration times of the two tested drugs.**

**
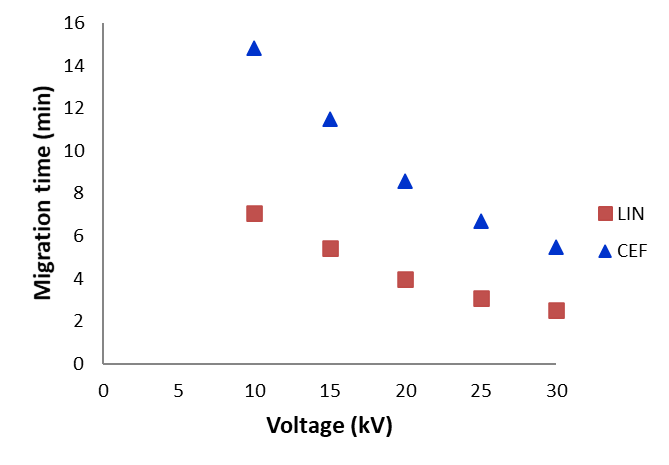
**

**Figure S3: Effect of applied voltage on migration times of the three tested drugs.**


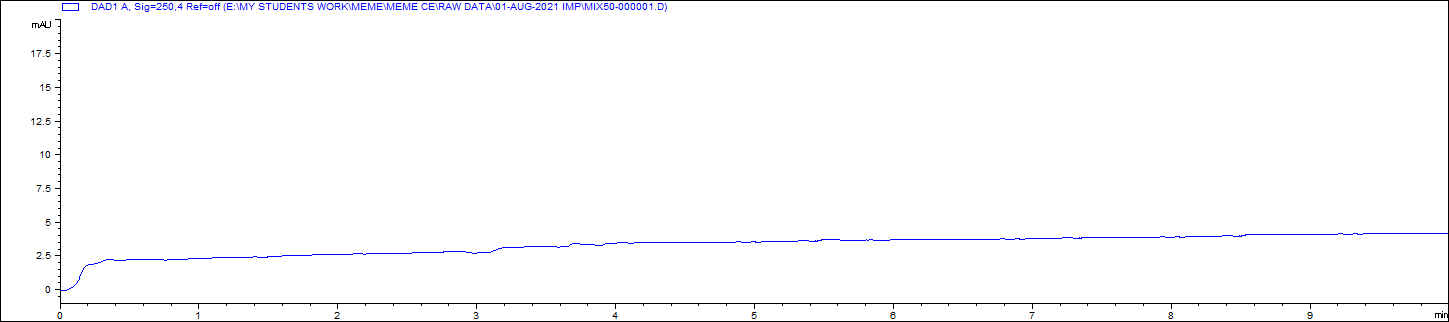


**Figure S4 : CZE electropherogram of a blank sample after extraction of a placebo laboratory prepared tablet that contains only the excipents without the two tested drugs.**

**Table S1: Effect of buffer concentration on peak hight, width, and symmetry**

| **Peak Symmetry** | | **Peak Width** | | **Peak Hight** | | **Buffer Concentration** |
| --- | --- | --- | --- | --- | --- | --- |
| **CEF** | **LIN** | **CEF** | **LIN** | **CEF** | **LIN** |  |
| 1.025 | 0.503 | 0.048 | 0.1112 | 10.5 | 5.1 | **25 mM** |
| 1.024 | 0.520 | 0.045 | 0.1093 | 15.7 | 7.1 | **50 mM** |
| 1.035 | 0.57 | 0.046 | 0.1073 | 16.7 | 7.8 | **75 mM** |
| 1.043 | 0.78 | 0.045 | 0.1041 | 19.6 | 8.6 | **100 mM** |

**Table S2: Comparison between the proposed CZE-DAD method with three previously reported methods regarding analytical performance.**

| **Comparison Points** | **The proposed CZE methods** | **The reported 1st Spectrophotometric method [39]^a^** | **The reported 2nd Spectrophotometric method [34]** | **The reported HPLC method [30]** |
| --- | --- | --- | --- | --- |
| **Linearity range in μg/mL** | CEF 5 – 50 μg/mL  LIN 5 – 50 μg/mL | CEF 1 – 20 μg/mL  LIN 1 – 20 μg/mL | CEF 2 – 20 μg/mL  LIN 5 – 20 μg/mL | CEF 1 – 5 μg/mL  LIN 3 – 15 μg/mL |
| **LOD ^a^** | CEF 0.301  LIN 1.213 | CEF 0.308, 0.281, 0.260  LIN 0.314, 0.254, 0.234 | CEF 0.347, 0.288, 0.368  LIN 0.193, 0.244, 0.114 | CEF 0.005  LIN 0.31 |
| **LOQ ^b^** | CEF 1.004  LIN 4.042 | CEF 0.932, 0.851, 0.787  LIN 0.95, 0.769, 0.710 | CEF 1.05, 0.87, 1.116  LIN 0.58, 0.74, 0.35 | CEF 0.015  LIN 0.93 |
| **Correlation coefficient** | CEF 0.9999  LIN 0.9999 | CEF ≥ 0.9997  LIN ≥ 0.9997 | CEF ≥ 0.9996  LIN ≥ 0.9995 | CEF 0.995  LIN 0.996 |
| **Sample volume per run** | 19.67 nL ^e^ | 3 mL | 3 mL | 20 µL |
| **Waste volume per run** | 19.67 nL | 3 mL | 3 mL | 8 mL |

^a^ Ref paper [39] has three different spectrophotometric methods.

^b^ Ref paper [34] has three different spectrophotometric methods.

^c^ Limit of detection in μg/mL

^d^ Limit of quantification in μg/mL

^e^ Calculated using CEToolbox [47]
